# VPS26A as a Prognostic Biomarker and Therapeutic Target in Liver Hepatocellular Carcinoma: Insights from Comprehensive Bioinformatics Analysis

**DOI:** 10.3390/medicina61071283

**Published:** 2025-07-16

**Authors:** Hye-Ran Kim, Jongwan Kim

**Affiliations:** 1 Department of Biomedical Laboratory Science, Dong-Eui Institute of Technology, 54 Yangji-ro, Busanjin-gu, Busan 47230, Republic of Korea; hrkim@dit.ac.kr; 2 Department of Anatomy, College of Medicine, Dongguk University, Gyeongju 38066, Republic of Korea

**Keywords:** LIHC, prognostic biomarker, TIICs, miRNA, bioinformatics analysis

## Abstract

*Background and Objectives:* VPS26A, a core component of the retromer complex, is pivotal to endosomal trafficking and membrane protein recycling. However, its expression profile, prognostic significance, and clinical relevance in liver hepatocellular carcinoma (LIHC) remain unexplored. This study investigates the prognostic potential of VPS26A by extensively analyzing publicly available LIHC-related databases. *Materials and Methods:* Multiple databases, including TIMER, UALCAN, HPA, GSCA, KM Plotter, OSlihc, MethSurv, miRNet, OncomiR, LinkedOmics, GeneMANIA, and STRING, were used to evaluate VPS26A expression patterns, prognostic implications, correlations with tumor-infiltrating immune cells (TIICs), epigenetic modifications, drug sensitivity, co-expression networks, and protein–protein interactions in LIHC. *Results:* VPS26A was significantly overexpressed at both the mRNA and protein levels in LIHC tissues compared to that in normal tissues. This upregulation was strongly associated with a poor prognosis. Furthermore, VPS26A expression was both positively and negatively correlated with various TIICs. Epigenetic analysis indicated that VPS26A is regulated by promoter and regional DNA methylation. Additionally, VPS26A influences the sensitivity of LIHC cells to a broad range of anticancer agents. Functional enrichment and co-expression analyses revealed that VPS26A serves as a central regulator of the LIHC transcriptomic landscape, with positively correlated gene sets linked to poor prognosis. Additionally, VPS26A contributes to the molecular architecture governing vesicular trafficking, with potential relevance to diseases involving defects in endosomal transport and autophagy. Notably, miRNAs targeting VPS26A-associated gene networks have emerged as potential prognostic biomarkers for LIHC. VPS26A was found to be integrated into a highly interconnected signaling network comprising proteins in cancer progression, immune regulation, and cellular metabolism. *Conclusions:* Overall, VPS26A may serve as a potential prognostic biomarker and therapeutic target in LIHC. This study provides novel insights into the molecular mechanisms underlying LIHC progression, and highlights the multifaceted role of VPS26A in tumor biology.

## 1. Introduction

Liver cancer represents a significant global health burden, ranking as the sixth most diagnosed malignancy and the fourth leading cause of cancer-related mortality worldwide [1,2]. Liver hepatocellular carcinoma (LIHC), the predominant histological subtype of primary liver cancer, accounts for approximately 75–85% of cases. The clinical outcomes of patients with LIHC are highly heterogeneous, largely owing to its multifactorial etiology. The well-established risk factors include chronic hepatitis B or C viral infections, alcohol consumption, tobacco use, metabolic disorders, and environmental carcinogens [3,4,5,6]. The heterogeneity of LIHC, driven by diverse pathogenic factors, hinders accurate prognostication, highlighting the need for more precise models that incorporate molecular and immune-related characteristics [7,8,9]. Although high-throughput sequencing and bioinformatics have enabled the discovery of various biomarkers, their clinical application remains limited, underscoring the need to integrate multidimensional molecular data.

Vacuolar protein sorting-associated protein 26A (VPS26A) is a core component of the retromer complex, a conserved multimeric protein assembly that mediates the retrograde trafficking of transmembrane proteins from endosomes to the trans-Golgi network. This process is essential for sorting and recycling various cargo proteins and is tightly linked to intracellular signaling, membrane homeostasis, and cellular differentiation processes [10,11,12,13]. Previous studies on VPS26A have primarily focused on its physiological and pathological roles in the nervous system. VPS26A facilitates the transition from stemness to differentiation in embryonic stem cells through the Nox4/ROS/ERK1/2 signaling cascade [14]. Moreover, decreased VPS26A expression has been observed in Alzheimer’s disease, and its dysregulation contributes to abnormal amyloid precursor protein processing and tau phosphorylation, implicating it in the pathogenesis of neurodegeneration [15,16]. Despite the increasing evidence implicating VPS26A in neurobiology, its role in tumorigenesis remains poorly understood. Few studies have investigated the involvement of VPS26A in cancer development. A recent integrative bioinformatics analysis identified VPS26A as a potential prognostic marker for LIHC [17]. However, comprehensive investigations of the expression patterns, prognostic significance, immune microenvironment associations, epigenetic regulation, and functional networks of VPS26A in cancer remain lacking. Given the crucial role of intracellular trafficking in oncogenic signaling, immune evasion, and therapeutic resistance, elucidating the role of VPS26A in cancer may offer novel insights into tumor biology and help identify new targets for intervention. This study aimed to systematically explore the expression landscape and clinical relevance of VPS26A in LIHC, specifically focusing on its prognostic value, correlation with immune infiltration, epigenetic modification, drug sensitivity, and the underlying molecular network. We propose that VPS26A functions as a regulator of tumor progression in LIHC, and may serve as a valuable biomarker for prognosis and therapeutic response.

The immune system plays a crucial role in modulating cancer progression [18]. Previous studies have demonstrated that tumor-infiltrating immune cells (TIICs) can aid the host in countering the development of cancer [19]. TIICs have emerged as a central focus in cancer research [20]. The density and classification of TIICs are significantly associated with the clinical outcomes of tumors and the efficacy of immunotherapy [21]. Numerous investigations have highlighted the characteristics of the immune response and its correlation with prognosis [22]. The prognostic relevance of TIICs has been emphasized in the context of LIHC.

DNA methylation plays a pivotal role in cancer onset and advancement, primarily by silencing tumor suppressor genes through promoter hypermethylation and activating various oncogenes via promoter hypomethylation [23,24,25,26]. Previous studies have indicated that during the pathogenesis of hepatitis B virus-related liver cancer development, the dysregulation of critical DNA methylation enzymes, including DNA methyltransferases, results in the silencing of tumor suppressor genes and the activation of oncogenes, thereby facilitating liver cancer progression [27,28]. Several studies have focused on identifying the DNA methylation biomarkers of LIHC [29,30,31]. Nevertheless, research examining the dynamic alterations in DNA methylation during LIHC progression remains limited.

Copy number variations (CNVs) in chromosomal segments, commonly referred to as aneuploidy, are prevalent in human cancers and have been identified as critical factors in the mechanisms underlying tumorigenesis [32,33]. Recent findings indicate that the loads of broad and focal CNVs exhibit differential correlations with gene expression markers associated with the key characteristics of cancer, including cell proliferation and immune evasion [34,35]. The interaction between the cancer genome and immune system may be influenced by a general gene dosage imbalance that governs specific genetic alterations. However, none of the cited studies, nor any other published research, have specifically investigated this phenomenon in the context of LIHC. This suggests that these alterations contribute to carcinogenesis through distinct mechanisms. Recent studies have indicated that the strength and orientation of the relationship between CNVs and tumor immunity may not be consistent across all cancer types [36,37,38]. This highlights the necessity of examining the effects of aneuploidy within the specific context of tumor tissues.

MicroRNAs (miRNAs) are small, endogenous, noncoding, single-stranded RNA molecules that play crucial roles in regulating the expression of target genes at both the post-transcriptional and translational stages. Dysregulated expression of miRNAs has been commonly identified in genomic regions associated with cancer or in fragile sites across various human cancers, including LIHC, where such alterations have been noted in both cellular and tissue contexts [39]. miRNAs have been associated with tumor prognosis, tumorigenesis, and tumor suppressors, as supported by several studies [40,41,42]. Research indicates that miRNAs display dysregulated expression patterns in LIHC and play a significant role in its growth, development, and metastasis by acting as either oncogenes or tumor suppressors [43]. Nevertheless, the underlying mechanisms and relationships between miRNAs and the pathogenesis of LIHC across different etiological contexts remain unclear [44].

Drug therapy remains a cornerstone in the treatment of numerous patients with cancer, as it can markedly enhance survival rates and elevate quality of life [45,46]. Recently, an improved understanding of the molecular biology of hepatocarcinogenesis and rapid advancements in diagnostic techniques have led to the approval of multiple drugs for advanced LIHC. Drug sensitivity tests have been successfully used in assay-guided chemotherapy for certain cancers. Nevertheless, the emergence of resistance to antineoplastic agents owing to genetic mutations and various nongenetic factors poses a significant challenge that restricts the effectiveness of treatment [47,48,49,50]. Increasing clinical evidence indicates that both intrinsic and acquired drug resistance in tumors is linked with genetic and epigenetic modifications.

In the present study, we comprehensively analyzed VPS26A expression in LIHC. Using an integrative bioinformatics approach, we evaluated VPS26A expression across various cancer types and confirmed its significant upregulation in LIHC at both mRNA and protein levels. Furthermore, we investigated the prognostic significance of VPS26A expression and demonstrated its association with poor prognosis across multiple subgroups. To elucidate the biological role of VPS26A in LIHC, we investigated its association with tumor-infiltrating immune cells and identified distinct immunological patterns, suggesting its involvement in modulating the tumor immune microenvironment. We found that VPS26A expression was influenced by both promoter and regional DNA methylation, underscoring its complex epigenetic regulation. Drug sensitivity profiling suggested that VPS26A could serve as a predictive biomarker of therapeutic responses. Further, co-expression and functional enrichment analyses demonstrated that VPS26A was closely associated with several oncogenic signaling pathways, including the PI3K/AKT and Wnt cascades. Collectively, these findings reveal that VPS26A is consistently overexpressed in LIHC and is functionally associated with tumor progression, immune regulation, and therapeutic responsiveness. We suggest that VPS26A functions as a multifaceted regulator of LIHC and has the potential as a biomarker for its diagnosis, prognosis, and therapeutic stratification. Overall, this study established a foundational framework for subsequent mechanistic studies and emphasized the potential clinical significance of targeting VPS26A for treating LIHC.

## 2. Materials and Methods

### 2.1. mRNA Expression Analysis of VPS26A in LIHC

To investigate the mRNA expression levels of VPS26A in LIHC, several publicly available bioinformatics databases were used, including TIMER2.0, GSCA (Gene Set Cancer Analysis), and UALCAN. These platforms integrate multi-omics datasets from The Cancer Gene Atlas (TCGA) [51,52,53,54]. Expression differences between LIHC and normal tissues were assessed using TIMER2.0 (http://timer.cistrome.org/) (accessed on 7 May 2025), which provides standardized gene expression estimates across cancer types. Further analyses were conducted using GSCA (http://bioinfo.life.hust.edu.cn/GSCA/#/) (accessed on 7 May 2025), a platform that integrates mRNA expression, methylation, immune cell infiltration, and survival data [55]. Expression levels across clinicopathological subgroups were analyzed using UALCAN (http://ualcan.path.uab.edu/) (accessed on 7 May 2025), a user-friendly portal that enables subgroup-specific analysis [56].

### 2.2. Protein Expression Analysis via Immunohistochemistry (IHC) of VPS26A in LIHC

Protein-level validation of VPS26A was performed using IHC data from the Human Protein Atlas (HPA) (https://www.proteinatlas.org) (accessed on 7 May 2025), which provides proteomic evidence across various cancer types [57,58]. IHC images of the normal liver and LIHC tissues were evaluated for staining intensity and cellular localization.

### 2.3. Prognostic Analysis of VPS26A in LIHC

To assess the prognostic significance of VPS26A, Kaplan–Meier (KM) survival curves were generated using KM Plotter (http://kmplot.com/analysis/) (accessed on 8 May 2025), GSCA, OSlihc (https://bioinfo.henu.edu.cn/LIHC/LIHCList.jsp) (accessed on 8 May 2025), and PrognoScan (http://dna00.bio.kyutech.ac.jp/PrognoScan/) (accessed on 8 May 2025) [59,60]. The evaluated survival endpoints included overall survival (OS), progression-free interval (PFI), disease-free interval (DFI), and disease-specific survival (DSS).

### 2.4. Immune Infiltration and Drug Sensitivity Analysis of VPS26A in LIHC

The relationship between VPS26A expression and immune cell infiltration was analyzed using GSCA, which incorporates precomputed immune cell scores derived from TCGA cohorts. Drug response data were retrieved from the Cancer Therapeutics Response Portal (CTRP) and Genomics of Drug Sensitivity in Cancer (GDSC) databases integrated within GSCA, enabling correlation analyses between gene expression and drug half-maximal inhibitory concentration (IC_50_) values [61,62].

### 2.5. DNA Methylation and Prognostic Analysis of VPS26A in LIHC

The methylation status of VPS26A was evaluated using OncoDB (https://oncodb.org/) (accessed on 10 May 2025), UALCAN, and MethSurv (https://biit.cs.ut.ee/methsurv/) (accessed on 10 May 2025) [63]. OncoDB was used to examine methylation profiles across normal and tumor tissues. UALCAN provided subgroup-specific methylation comparisons and MethSurv enabled methylation–survival correlation analysis.

### 2.6. Gene–Chemical Interaction Analysis of VPS26A

To explore the interactions between VPS26A and chemicals, the Comparative Toxicogenomics Database (CTD) (http://ctdbase.org/) (accessed on 10 May 2025) was used. CTD provides curated datasets that link gene expression with environmental chemical exposures and drug interactions [64].

### 2.7. Co-Expressed Genes and Functional Enrichment Analysis Associated with VPS26A in LIHC 

Gene co-expression and enrichment analyses were conducted using LinkedOmics (http://www.linkedomics.org) (accessed on 10 May 2025), a TCGA-based platform that provides expression correlation and Gene Set Enrichment Analysis (GSEA) functions [65]. Gene Ontology (GO) and Kyoto Encyclopedia of Genes and Genomes (KEGG) pathway enrichment analyses were performed using 500 permutations, with a false discovery rate (FDR) < 0.05 considered significant.

### 2.8. Prognostic Analysis of Co-Expressed Genes Associated with VPS26A in LIHC

The GEPIA2 platform (http://gepia2.cancer-pku.cn) (accessed on 11 May 2025) was used to determine the prognostic impact of genes co-expressed with VPS26A in LIHC [66]. Genes identified from LinkedOmics were input into “Survival Map” in GEPIA2 to evaluate correlations with OS and DFS.

### 2.9. Gene–Gene Interaction (GGI) Network Analysis of VPS26A in LIHC

GeneMANIA (http://www.genemania.org) (accessed on 11 May 2025) was used to construct the GGI network involving VPS26A and its interactors. GeneMANIA integrates data from physical interaction, co-expression, and genetic interaction datasets [67].

### 2.10. miRNA Network and Prognostic Analysis of VPS26A-Related Genes in LIHC

miRNAs that potentially regulate VPS26A-related genes were identified using miRNet (https://www.mirnet.ca) (accessed on 11 May 2025) [68]. The OncomiR platform (https://oncomir.org) (accessed on 12 May 2025) was used to determine the prognostic relevance of these miRNAs in LIHC [69].

### 2.11. Protein–Protein Interaction (PPI) Networks and Enrichment of VPS26A

Protein–protein interaction networks for VPS26A were constructed using the STRING database (https://string-db.org) (accessed on 13 May 2025), which integrates both experimentally validated and predicted PPIs [70]. GO and KEGG enrichment analyses were conducted to assess the functional relevance of VPS26A and its protein partners.

### 2.12. Statistical Analysis

Expression comparisons between tumor and normal tissues were performed using built-in tools from TIMER2.0, UALCAN, and OncoDB. Survival analyses (KM curves and log-rank tests) were performed using GSCA, GEPIA2, MethSurv, and OncomiR. Cox proportional hazards regression models were used to estimate the hazard ratios (HRs) and 95% confidence intervals. Pearson correlation and FDR correction were applied to control for multiple testing, with *p* < 0.05 or FDR < 0.05 considered statistically significant.

## 3. Results

### 3.1. mRNA Expression of VPS26A in LIHC

**Figure 1:**
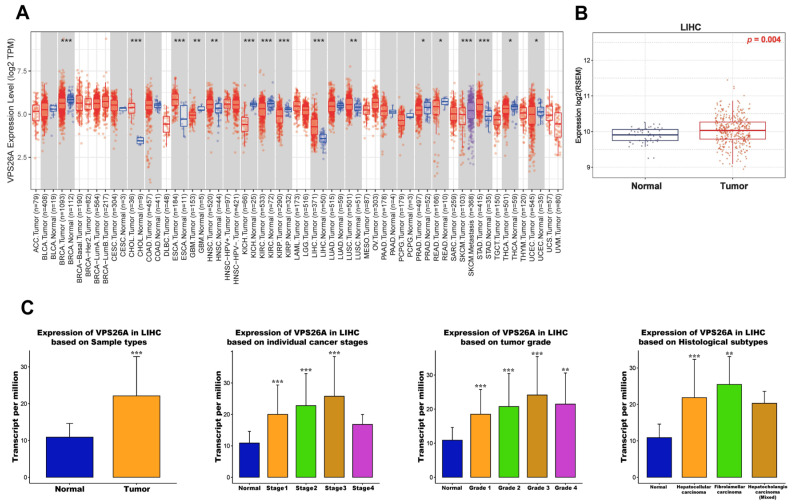
mRNA expression levels of VPS26A in LIHC. (**A**) Comparison of VPS26A expression between tumor and normal tissues in various cancer types. (**B**) Comparison of VPS26A expression between LIHC and normal liver tissue. (**C**) Comparison of VPS26A expression between clinicopathologic characteristics and normal conditions. * *p* < 0.05, ** *p* < 0.01, and *** *p* < 0.001.

To explore the expression profile of VPS26A across various cancer types, we performed a pan-cancer analysis using RNA sequencing data from TCGA via the TIMER database. As shown in Figure 1A, VPS26A expression was significantly elevated in multiple cancers, such as liver hepatocellular carcinoma (LIHC), breast invasive carcinoma (BRCA), bladder cholangiocarcinoma (CHOL), esophageal carcinoma (ESCA), head and neck squamous cell carcinoma (HNSC), kidney renal clear cell carcinoma (KIRC), lung squamous cell carcinoma (LUSC), and stomach adenocarcinoma (STAD), compared to that in the corresponding normal tissues. In contrast, VPS26A expression was significantly downregulated in several tumor types, including glioblastoma multiforme (GBM), kidney chromophobe (KICH), kidney renal papillary cell carcinoma (KIRP), prostate adenocarcinoma (PRAD), rectal adenocarcinoma (READ), thyroid carcinoma (THCA), and uterine corpus endometrial carcinoma (UCEC). To further validate VPS26A expression in LIHC, we used the GSCA database, which confirmed a significant upregulation of VPS26A expression in LIHC compared to that in normal tissues (Figure 1B). As shown in Figure 1C, VPS26A expression in LIHC was evaluated using the UALCAN database, which enabled assessment across clinical subgroups. VPS26A expression was found to correlate significantly with several clinical parameters, including primary tumor; tumor stages I, II, and III; tumor grades I, II, III, and IV; and histological subtypes. Collectively, these findings reinforce that VPS26A is markedly upregulated in LIHC across diverse clinicopathological conditions. The overexpression observed in these databases demonstrates the potential importance of VPS26A as a pan-cancer biomarker with diagnostic and prognostic significance in LIHC.

### 3.2. Protein Expression of VPS26A in LIHC

**Figure 2:**
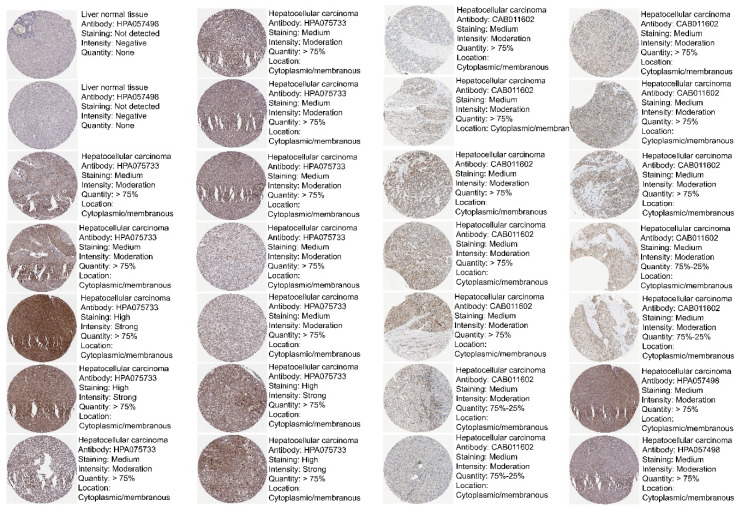
Protein expression levels of VPS26A in LIHC. Protein expression of VPS26A in immunohistochemical images of LIHC.

To evaluate the protein expression levels of VPS26A in LIHC, we performed IHC analysis using data from the HPA. The IHC images are shown in Figure 2. This analysis indicated that VPS26A protein expression was undetectable in normal tissues. LIHC demonstrated marked upregulation of VPS26A protein expression, as indicated by strong cytoplasmic staining in tumor cells. The results indicated an elevation in the protein levels of VPS26A in LIHC tissues compared to those in normal tissues. These findings demonstrate that VPS26A is consistently upregulated at both the mRNA and protein levels in LIHC, thereby supporting its potential utility as a diagnostic and prognostic biomarker for LIHC.

### 3.3. Prognostic Value of VPS26A Expression in LIHC

**Figure 3:**
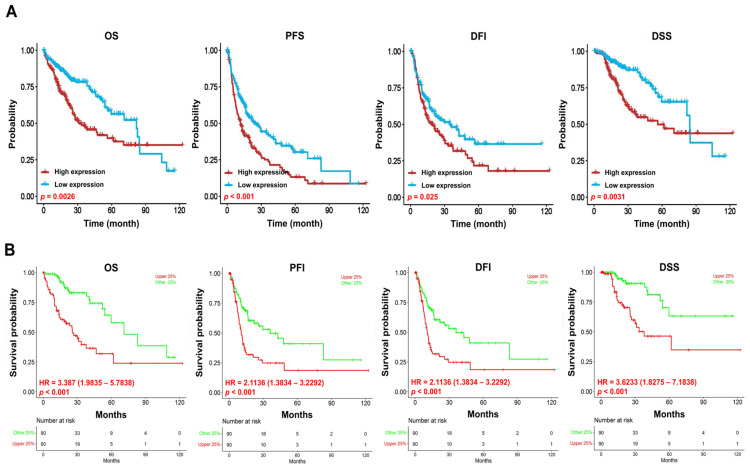
Prognostic significance of VPS26A expression in LIHC. The prognostic value of VPS26A expression was analyzed using GSCA (**A**) and OSlihc (**B**). Overall survival, OS; progression-free survival, PFS; progression-free interval, PFI; disease-free interval, DFI, disease-specific survival, DSS.

To assess the prognostic value of VPS26A expression in LIHC, we performed survival analyses using the KM Plotter, OSlihc, and GSCA databases. Elevated VPS26A expression was found to be associated with poor survival outcomes. Elevated VPS26A expression was significantly associated with poor prognoses, including overall survival (OS; *p* = 0.0026), progression-free survival (PFS; *p* < 0.001), disease-free interval (DFI; *p* = 0.025), and disease-specific survival (DSS; *p* = 0.0031) (Figure 3A). Further analysis revealed that elevated VPS26A expression was significantly associated with poor prognosis in terms of OS (HR = 3.387, *p* < 0.001), PFI (HR = 2.1136, *p* < 0.001), DFI (HR = 2.1136, *p* < 0.001), and DSS (HR = 3.6233, *p* < 0.001) (Figure 3B). In exploring correlations with clinicopathological characteristics using the KM plotter database, elevated VPS26A expression was associated with poor prognosis across multiple patient subsets, including both male (HR = 1.7, *p* = 0.02) and female (HR = 2.55, *p* = 0.0019) patients, earlier stages (stage I: HR = 2.38, *p* = 0.0065; stages I and II: HR = 2.34, *p* = 0.0007), higher grades (grade III: HR = 2.43, *p* = 0.0047), AJCC_ tumors (AJCC_T I: HR = 2.4, *p* = 0.0042), absence of vascular invasion (HR = 2.7, *p* = 0.0003), white (HR = 2.23, *p* = 0.00075) and Asian (HR = 2.0, *p* = 0.025) patients, patients without alcohol consumption (HR = 1.96, *p* = 0.0051), and patients with or without a hepatitis virus infection (HR = 2.04, *p* = 0.038 and HR = 2.09, *p* = 0.00022, respectively) (Table 1). Moreover, analysis across various cancer types further supported the association between elevated VPS26A expression and adverse survival outcomes (Table 2). Collectively, these findings indicate that elevated VPS26A expression serves as a robust marker of poor prognosis in LIHC, emphasizing its potential as a significant prognostic biomarker.

### 3.4. Correlation Between VPS26A and TIICs in LIHC

**Figure 4:**
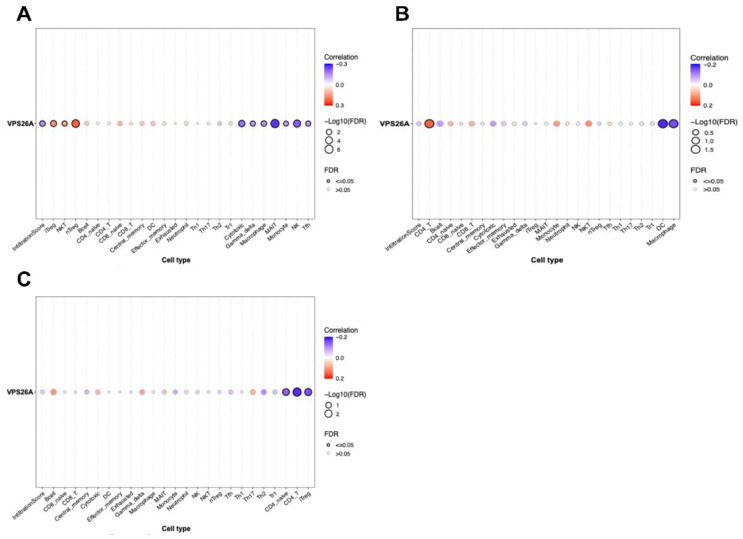
Correlation between VPS26A expression and TIICs in LIHC. The correlation between VPS26A and tumor-infiltrating immune cells (TIICs) using the GSCA database. Expression of VPS26A (**A**), copy number variation (CNV) of VPS26A (**B**), methylation of VPS26A (**C**).

**Figure 5:**
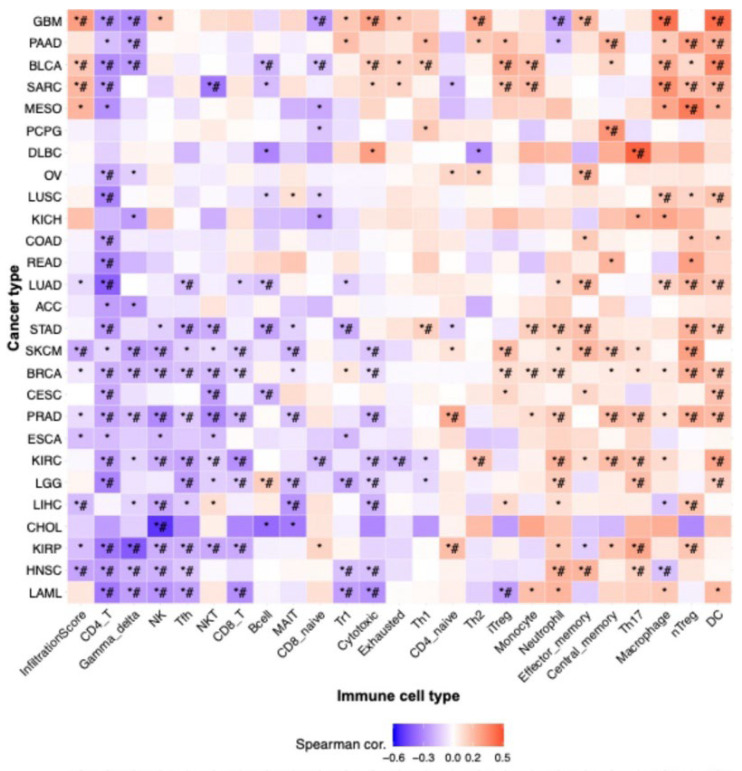
Correlation between VPS26A expression and tumor-infiltrating immune cells (TIICs) in various cancers. The correlation between VPS26A and TIICs in various cancers using the GSCA database. * *p*-value ≤ 0.05, # FDR ≤ 0.05.

To explore the immunological landscape associated with VPS26A expression in LIHC, we performed immune infiltration analysis using the GSCA database. As shown in Table 3 and Figure 4, VPS26A expression was positively correlated with the infiltration levels of several immune cell types, including natural regulatory T (nTreg) cells (R = 0.23, *p* < 0.001), induced regulatory T (iTreg) cells (R = 0.17, *p* < 0.001), natural killer T (NKT) cells (R = 0.13, *p* < 0.001), and naïve CD8 + T cells (R = 0.11, *p* = 0.01) (Figure 4A). Conversely, VPS26A expression demonstrated a negative correlation with mucosal-associated invariant T (MAIT) cells (R = −0.26, *p* < 0.001), natural killer (NK) cells (R = −0.21, *p* < 0.001), cytotoxic T cells (R = −0.19, *p* < 0.001), macrophages (R = −0.15, *p* = 0.004), gamma delta T cells (R = −0.14, *p* ≤ 0.005), follicular helper T (Tfh) cells (R = −0.13, *p* = 0.008), and monocytes (R = −0.12, *p* = 0.001). Analysis of copy number variations (CNVs) in VPS26A revealed further immune correlations (Figure 4B). The CNVs of VPS26A were positively associated with CD4 + T cells (R = 0.15, *p* = 0.004), NKT cells (R = 0.12, *p* = 0.02), and monocytes (R = 0.11, *p* = 0.04). In contrast, the CNVs of VPS26A showed a negative correlation with dendritic cells (DCs) (R = −0.18, *p* = 0.001), and macrophages (R = −0.16, *p* = 0.003). VPS26A methylation was also assessed in relation to immune cell infiltration (Figure 4C). Methylation of VPS26A positively correlated with B cell infiltration (R = 0.11, *p* = 0.02), whereas negative correlations were observed with CD4 + T cells (R = −0.18, *p* = 0.001), iTreg cells (R = −0.15, *p* = 0.003), and naïve CD4 + T cells (R = −0.14, *p* = 0.008). GSEA was performed to determine immune-related enrichment scores (ESs) associated with VPS26A expression across 33 cancer types. In LIHC, VPS26A was positively correlated with the infiltration of NKT cells, iTreg cells, nTreg cells, and neutrophils. Conversely, a negative correlation was noted between gamma delta T cells, NK cells, Tfh cells, MAIT cells, and cytotoxic T cells (Figure 5), suggesting that VPS26A expression is closely linked to the tumor immune microenvironment in LIHC.

### 3.5. Correlation of VPS26A Expression with DNA Methylation in LIHC

**Figure 6:**
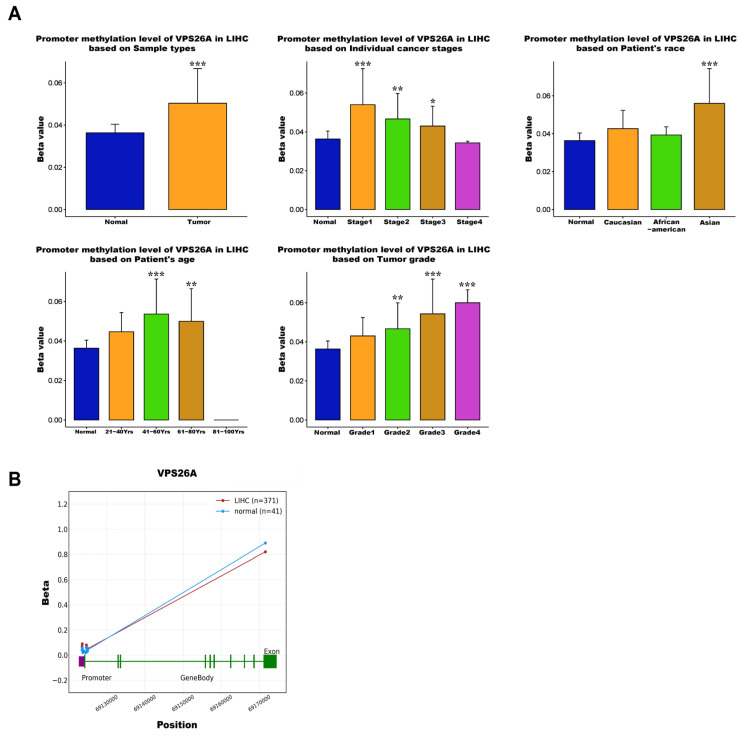
Correlation between VPS26A expression and DNA methylation in LIHC. (**A**) The correlation between VPS26A and DNA methylation. (**B**) The correlation between VPS26A and DNA methylation in tumor and normal tissues. * *p* < 0.05, ** *p* < 0.01, and *** *p* < 0.001.

**Figure 7:**
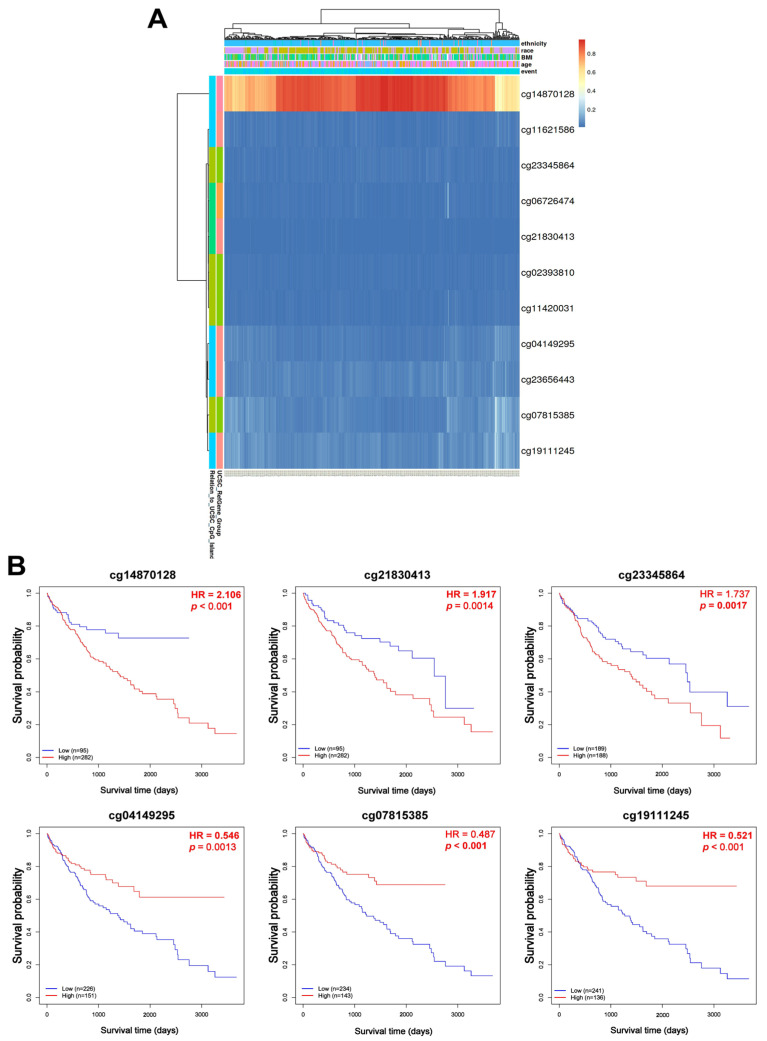
Prognostic significance of DNA methylation of VPS26A in LIHC. (**A**) The heat map for promoter methylation of VPS26A expression. (**B**) The KM plots for promoter methylation of VPS26A expression.

To investigate the regulatory role of DNA methylation in VPS26A expression, analyses were conducted using the UALCAN and OncoDB databases. The results revealed that VPS26A expression was significantly influenced by promoter hypomethylation in LIHC. VPS26A expression was notably affected by clinical parameters such as the primary tumor, race (Asian), age group (41–60 and 61–80 years), tumor stage (I, II, and III), and tumor grade (II, III, and IV) (Figure 6A). Further analysis indicated that VPS26A expression was associated with the methylation levels of specific probes distributed across both the promoter and exon regions in tumor and normal tissues. (Figure 6B and Table 4). Heat map visualization from the MethSurv database demonstrated differential methylation patterns, where hypermethylated sites are shown in red and hypomethylated sites are shown in blue (Figure 7A). Among them, the cg14870128 probe was identified within the hypermethylated region of the gene. Survival analysis was conducted to assess the prognostic relevance of methylation-associated probes of VPS26A. Notably, hypermethylated probes, such as cg14870128 (HR = 2.106, *p* < 0.001), cg21830413 (HR = 1.917, *p* = 0.0014), and cg23345864 (HR = 1.737, *p* = 0.0017), were significantly associated with poor prognosis in patients with LIHC. Interestingly, hypomethylated probes, including cg04149295 (HR = 0.546, *p* = 0.0013), cg07815385 (HR = 0.487, *p* < 0.001), and cg07815385 (HR = 0.521, *p* < 0.001), were also associated with poor prognosis in LIHC (Figure 7B). These findings suggest that VPS26A expression is associated with poor prognosis through both promoter and methylation changes in LIHC.

### 3.6. Correlation Between VPS26A Expression and Drug Sensitivity in LIHC

**Figure 8:**
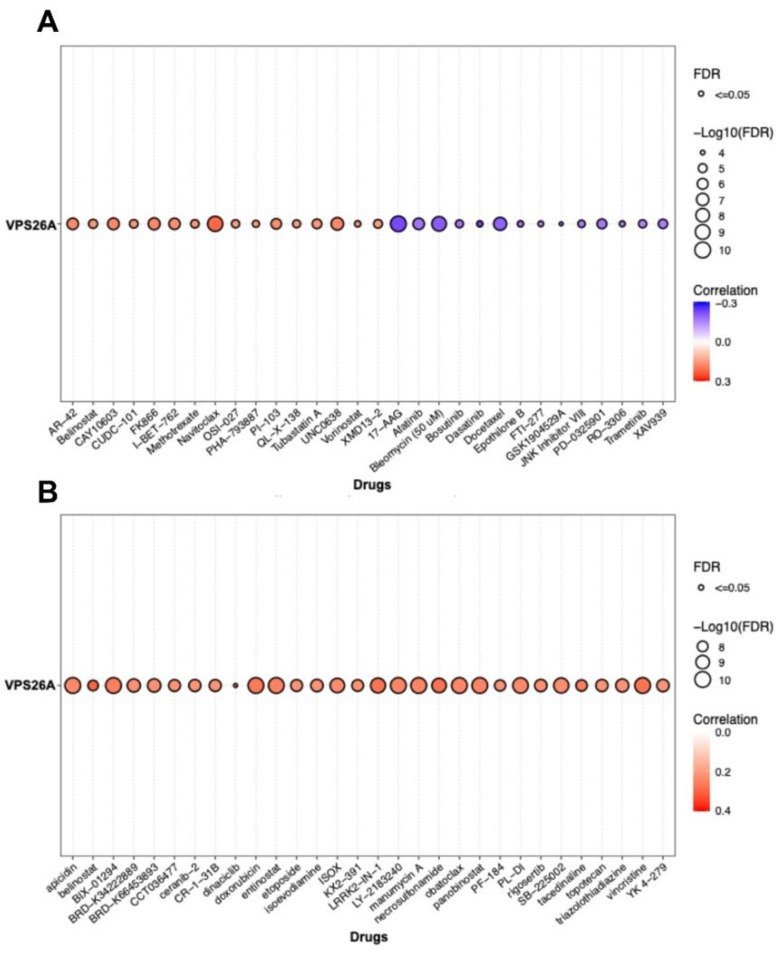
Correlation between VPS26A expression and drug sensitivity. (**A**) The correlation between VPS26A and drug sensitivity using GDSC in the GSCA database (**B**) The correlation between VPS26A and drug sensitivity using CTRP in the GSCA database.

To investigate the association between VPS26A expression and the drug response in LIHC, drug sensitivity analyses were performed using data from the GDSC and CTRP databases. GDSC dataset analysis indicated a positive and negative correlation between VPS26A expression and increased sensitivity to various anticancer agents. Specifically, AR-42, belinostat, CAY10603, CUDC-101, FK866, I-BET-762, methotrexate, navitoclax, OSI-027, PHA-793887, OU-103, QL-X-138, tubastatin, UNC0638, vorinostat, and XMD13-2 were positively correlated with VPS26A expression (Figure 8A). Conversely, a negative correlation was identified between VPS26A expression and sensitivity to drugs, including 17-AAG, afatinib, bleomycin, bosutinib, dasatinib, docetaxel, epothilone B, FTI-277, GSK1904529A, JNK inhibitor VIII, PD-0325901, RO-3306, trametinib, and XAV939. Supporting these findings, analysis of the CTRP dataset further corroborated the positive relationship between VPS26A expression and sensitivity to various drugs such as apicidin, belinostat, doxorubicin, and necrosulfonamide (Figure 8B). These results suggest that VPS26A significantly influences the cellular responses to various anticancer drugs, indicating its potential as a predictive biomarker for treatment sensitivity in LIHC.

### 3.7. Interactions Between Chemicals and VPS26A in LIHC

To explore the regulatory influence of chemical compounds on VPS26A expression, an analysis was conducted using data from the CTD. This analysis identified 82 chemicals associated with VPS26A, of which 59 were associated with the upregulation of VPS26A expression and 23 were linked to its downregulation (Table 5). Furthermore, the analysis identified the top 20 gene–chemical interactions involving VPS26A, which revealed significant correlations with several genes. Notably, VPS26A exhibited strong associations with genes such as VPS35 (vacuolar protein sorting 35), GCN1 (general control nonderepressible 1), VTI1B (vesicle transport through interaction with T-SNAREs homolog 1B), UFM1 (ubiquitin-fold modifier 1), and VPS29 (vacuolar protein sorting 29) (Table 6). These findings suggest that VPS26A is part of a chemically responsive network that involves key players in vesicular trafficking, stress responses, and protein sorting. Interactions between VPS26A, various chemicals, and functionally related genes underscore its potential role as a regulatory hub.

### 3.8. Co-Expression and Functional Enrichment Analysis of VPS26A in LIHC

**Figure 9:**
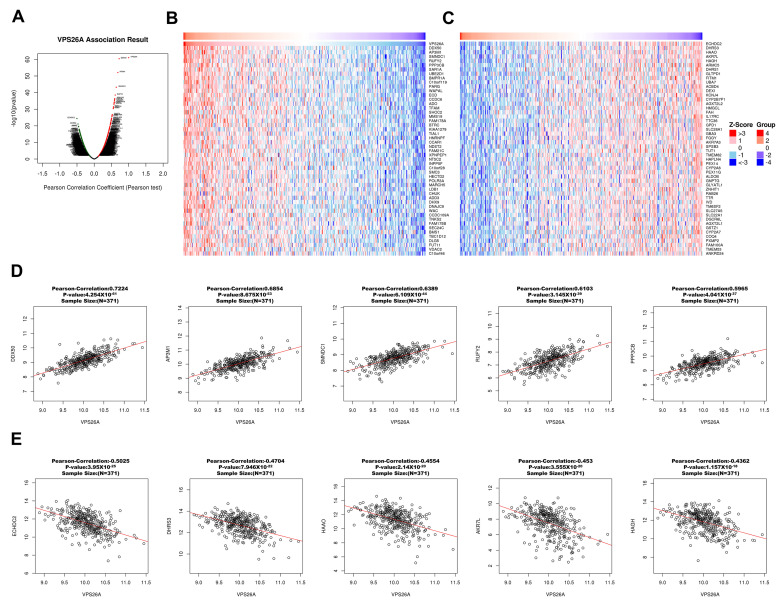

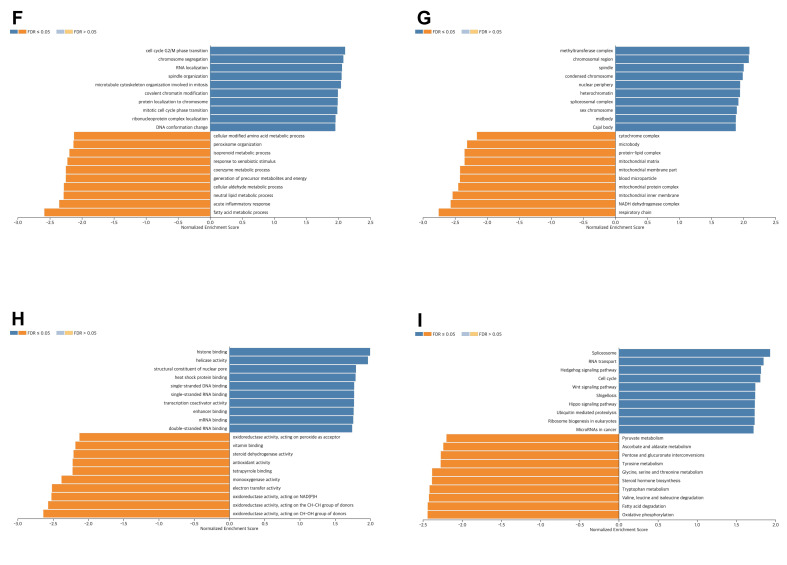
Co-expression and functional enrichment for VPS26A expression in LIHC. (**A**) The genes positively and negatively correlated with VPS26A expression in LIHC. (**B**) Heat map showing the top 50 genes positively correlated with VPS26A expression in LIHC. (**C**) Heat map showing the top 50 genes negatively correlated with VPS26A expression in LIHC. (**D**) The correlation of the top five genes positively correlated with VPS26A expression in LIHC. (**E**) The correlation of the top five genes negatively correlated with VPS26A expression in LIHC. (**F**) Enriched GO-biological processes related to the co-expressed genes of VPS26A by GSEA. (**G**) Enriched GO-molecular functions related to the co-expressed genes of VPS26A by GSEA. (**H**) Enriched GO-cellular components related to the co-expressed genes of VPS26A by GSEA. (**I**) Enriched GO-KEGG pathway related to the co-expressed genes of VPS26A by GSEA. Dark blue and orange indicate a false discovery rate (FDR) ≤ 0.05 and light blue and orange indicate FDR > 0.05.

A thorough co-expression analysis was performed using the LinkedOmics database to explore the potential biological functions of VPS26A in LIHC. This analysis revealed that 12,195 genes exhibited a positive correlation with VPS26A, as represented by dark red dots, whereas 7727 genes demonstrated a negative correlation, as indicated by dark green dots (Figure 9A). Heat map visualization was used to identify the top 50 genes showing the most significant positive and negative correlations with VPS26A expression (Figure 9B,C). Among these, the five genes with the highest positive correlations were DDX50 (R = 0.7224, *p* < 0.001), AP3M1 (R = 0.6854, *p* < 0.001), SMNDC1 (R = 0.6389, *p* < 0.001), RUFY2 (R = 0.6103, *p* < 0.001), and PPP3BX (R = 0.5965, *p* < 0.001) (Figure 9D). In contrast, notable negative correlations were found with ECHDC2 (R = −0.5025, *p* < 0.001), DHRS3 (R = −0.4704, *p* < 0.001), HAAO (R = −0.4554, *p* < 0.001), AKR7L (R = −0.453, *p* < 0.001), and HAGH (R = −0.4362, *p* < 0.001) (Figure 9E). GSEA of GO terms indicated that genes associated with VPS26A were significantly enriched in biological processes, such as G2/M phase transition, chromosome segregation, RNA localization, and spindle organization (Figure 9F). Molecular functions associated with methyltransferase complexes, chromosomal regions, spindle components, and condensed chromosomes were analyzed (Figure 9G). Cellular components associated with histone binding, helicase activity, nuclear pore complex structure, and heat shock protein binding were also analyzed (Figure 9H). Further pathway enrichment analysis via GSEA-KEGG revealed that VPS26A-co-expressed genes were predominantly involved in pathways such as the spliceosome, RNA transport, Hedgehog signaling, cell cycle regulation, and Wnt signaling (Figure 9I). These findings suggest that VPS26A plays a key regulatory role in the LIHC transcriptomic landscape.

### 3.9. Prognostic Value of VPS26A-Associated Genes in LIHC

**Figure 10:**
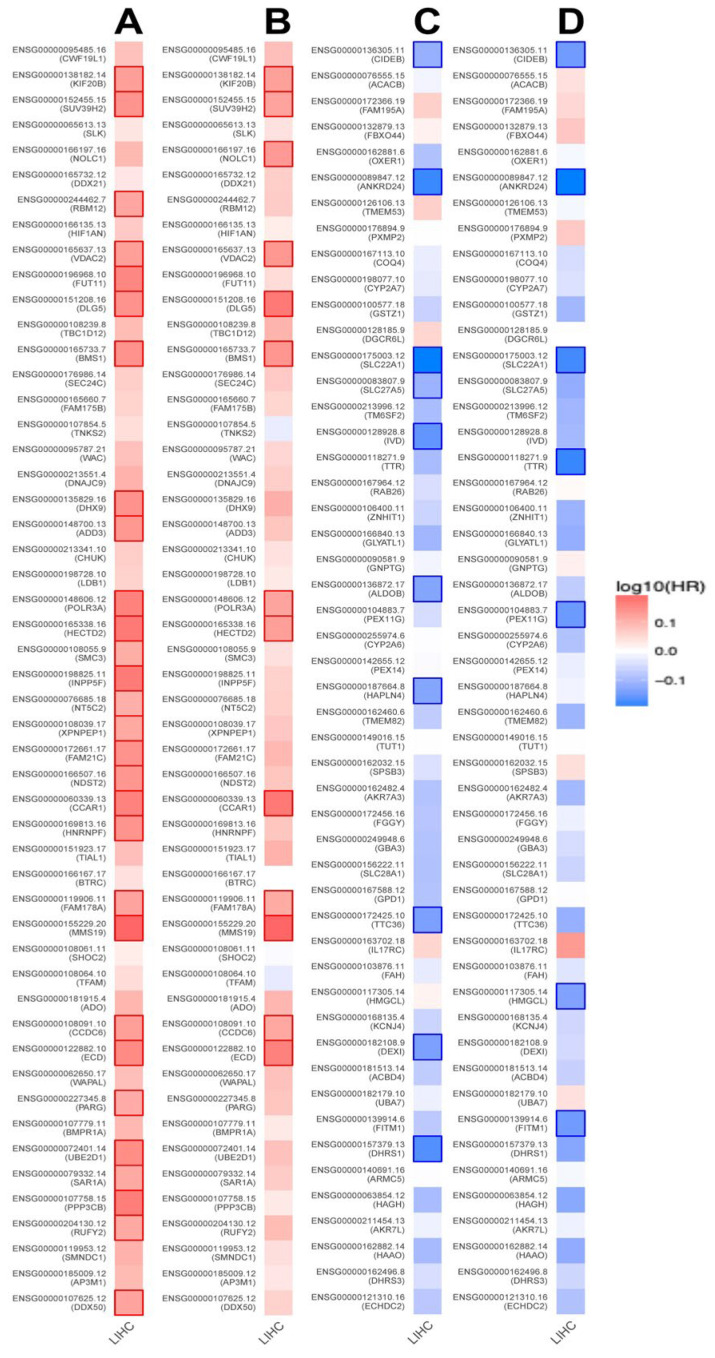
Prognostic significance of VPS26A-related genes in various cancer types including LIHC. Survival map of the positively correlated genes of VPS26A for OS (**A**) and DFS (**B**). Survival map of genes negatively correlated with VPS26A expression for OS (**C**) and DFS (**D**). Heat map showing the log_10_ (HR) of genes in LIHC. Squares with a bold border represent a *p*-value < 0.05 in the survival analysis.

To evaluate the prognostic significance of VPS26A-associated genes in LIHC, analysis was performed using the ZEPIA2 database. The results revealed that genes exhibiting a positive correlation with VPS26A may serve as potential high-risk factors for LIHC. Specifically, among the genes that positively correlated with VPS26A, 31 were associated with a high HR for OS (Figure 10A), whereas 13 demonstrated a high HR for DFS (Figure 10B). In contrast, genes that exhibited a negative correlation with VPS26A were associated with a more favorable prognostic profile. Notably, ten of these were linked to a low HR for OS (Figure 10C), while seven of these genes were linked to a low HR for DFS (Figure 10D). These results suggest that VPS26A and its positively correlated gene set are associated with a poor prognosis in patients with LIHC.

### 3.10. Gene–Gene Interaction (GGI) Network Analysis of VPS26A

**Figure 11:**
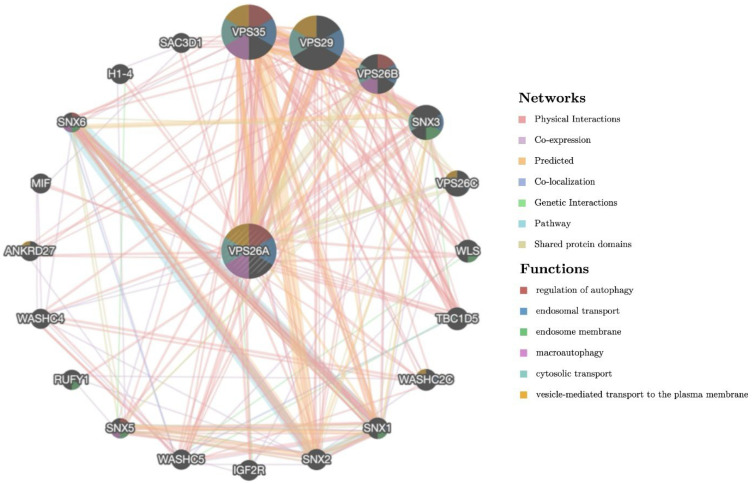
Gene–gene interaction (GGI) network analysis of VPS26A.

To explore the gene–gene interactions of VPS26A, the interacting genes of VPS26A were analyzed using GeneMANIA. The investigation generated a comprehensive network showing the significant interactions among the target genes as well as the associations with other functionally relevant genes. The results showed the 20 most frequently altered genes closely correlated with VPS26A, in which VPS35, VPS29, VPS26B, and SNX3 (sorting nexin 3) showed the most significant correlation with VPS26A. The analysis revealed that the core elements of the retromer complex, particularly VPS26A and VPS35, were strongly co-expressed and shared involvement in multiple overlapping biological pathways. These genes are significantly associated with key cellular processes, including the regulation of autophagy, macroautophagy, endosomal transport, cytosolic transport, and vesicle-mediated transport to the plasma membrane. These findings provide valuable insights into the molecular framework governing vesicular trafficking with potential implications for understanding disorders linked to defects in endosomal transport and autophagy (Figure 11).

### 3.11. Prognostic Value of miRNAs Targeting VPS26A-Associated Genes in LIHC

**Figure 12:**
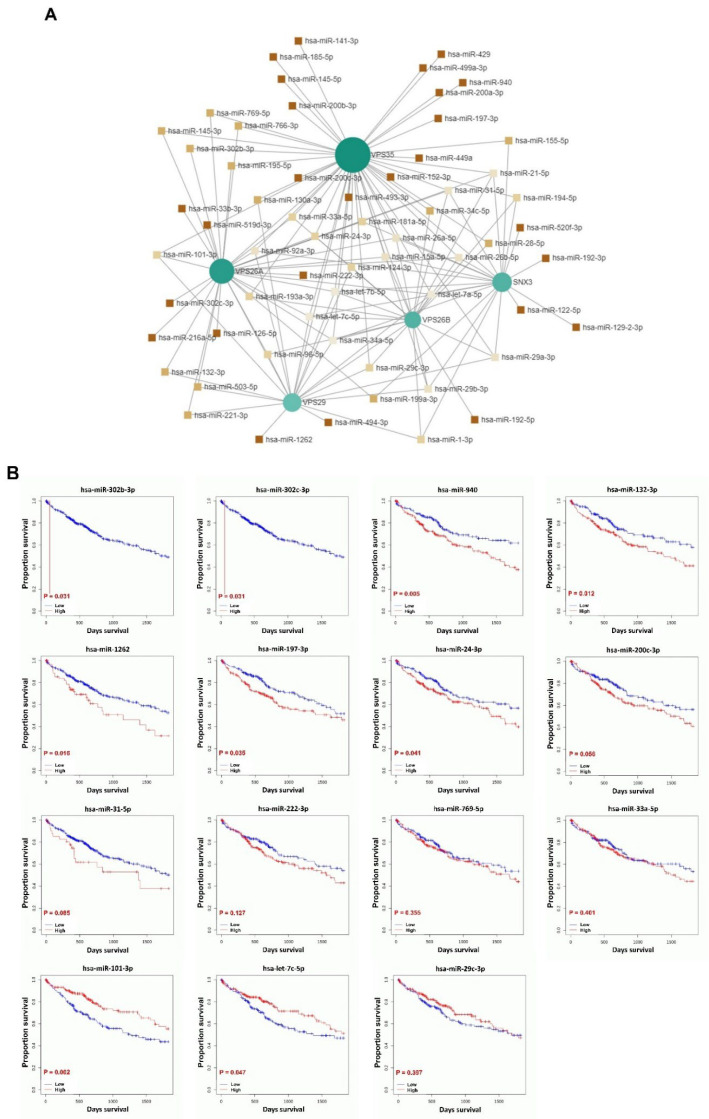
Prognostic significance of miRNAs in VPS26A-associated genes in LIHC. (**A**) The miRNA interaction network for VPS26A-associated genes. (**B**) Prognostic significance of miRNAs for VPS26A-associated genes.

To investigate miRNA interactions and the prognostic value of miRNAs targeting VPS26A-associated genes, an miRNA–gene interaction network was constructed using the miRNet database. This analysis identified 61 miRNAs that target VPS26A-associated genes (Figure 12A and Table 7). Survival analyses were performed using the OncomiR database to assess the prognostic relevance of these miRNAs. Of 61 miRNAs, 15 were significantly associated with a poor prognosis in LIHC. These included hsa-miR-302b-3p, hsa-miR-302c-3p, hsa-miR-940, hsa-miR-132-3p, hsa-miR-1262, hsa-miR-197-3p, hsa-miR-24-3p, hsa-miR-200c-3p, hsa-miR-31-5p, hsa-miR-222-3p, hsa-miR-769-5p, hsa-miR-33a-5p, hsa-miR-101-3p, hsa-miR-let-7c-5p, and hsa-miR-29c-3p. Among these, hsa-miR-302b-3p, hsa-miR-302c-3p, hsa-miR-940, hsa-miR-132-3p, hsa-miR-1262, hsa-miR-197-3p, and hsa-miR-24-3p were associated with a poor prognosis when highly expressed in LIHC (Figure 12B). In contrast, hsa-miR-101-3p and hsa-miR-let-7c-5p were associated with a poor prognosis when expressed at low levels in LIHC. These findings suggest that miRNAs targeting VPS26A-related gene networks may serve as valuable prognostic biomarkers for LIHC.

### 3.12. Protein–Protein Interaction and Functional Enrichment Analysis of VPS26A in LIHC

**Figure 13:**
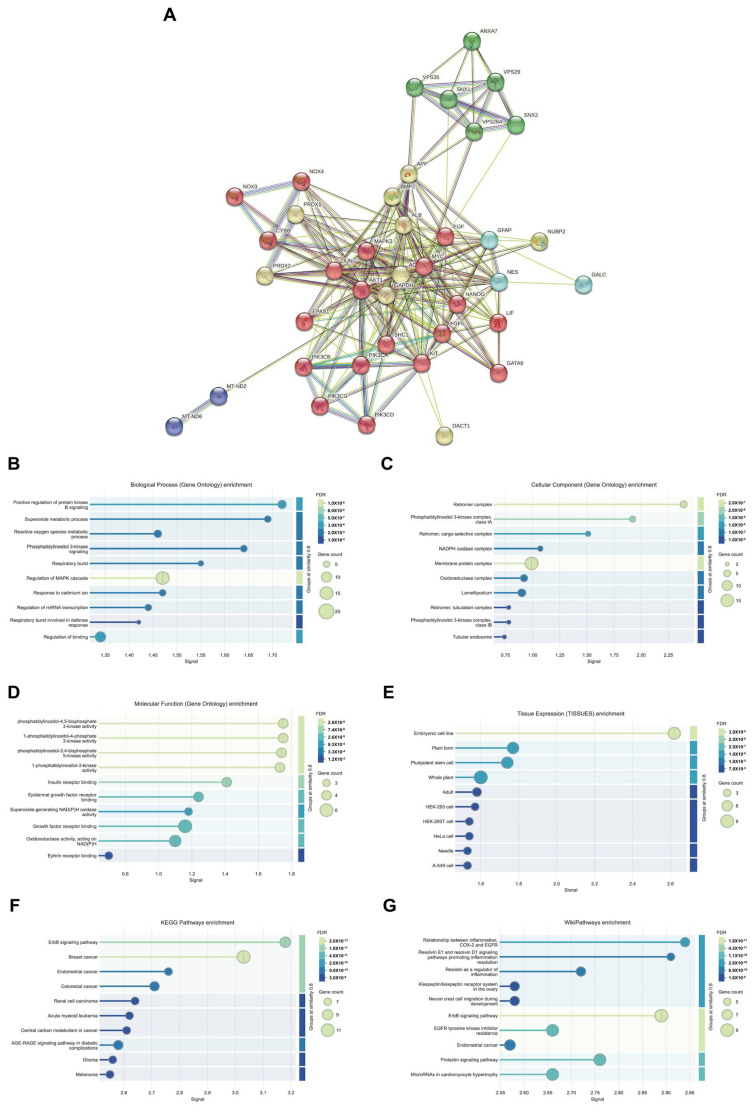
Protein–protein interaction and functional enrichment of VPS26A expression. (**A**) Protein–protein interaction of VPS26A expression. (**B**) Enriched GO biological processes related to co-expressed proteins of VPS26A. (**C**) Enriched GO cellular components related to co-expressed proteins of VPS26A. (**D**) Enriched GO molecular functions related to co-expressed proteins of VPS26A. (**E**) Enriched GO tissue expression related to co-expressed proteins of VPS26A. (**F**) Enriched GO-KEGG pathway related to co-expressed genes of VPS26A by GSEA. (**G**) Enriched GO-Wiki pathway related to co-expressed genes of VPS26A by GSEA.

To elucidate the protein–protein interaction (PPI) of VPS26A, we constructed a PPI network using the STRING database. The resulting network comprised 234 edges and 39 nodes, illustrating the complex interaction architecture associated with VPS26A (Figure 13A). PPI network analysis revealed five distinct functional clusters, including an ErbB/PI3K-AKT Signaling Cluster, which is a major cluster of 19 genes including LIF, GATA6, NANOG, FGF5, EGF, MYC, EPAS1, KIT, AKT1, JUN, MAPK3, and multiple PIK3 subunits, associated with ErbB signaling and PI3K/AKT signaling regulation; a neurodegenerative and oxidative stress cluster, which is a second cluster containing 9 genes (DACT1, GAPDH, APP, ACTB, ALB, BMP2, NUBP2, PRDX1, and PRDX2) linked to oxidative stress and neurodegenerative pathways, particularly those related to Alzheimer’s disease; a leukodystrophy-associated cluster, including the 3 genes NES, GFAP, and GALC which are associated with leukodystrophy-related processes; and the mitochondrial dysfunction cluster, including MT-ND2 and MT-ND6 which are linked to optic neuropathy and ubiquinone metabolism. In addition, we analyzed biological processes through GO and pathway enrichment. PPI-related proteins were significantly enriched in these processes, including the positive regulation of protein kinase B (AKT) signaling, the superoxide metabolic process, phosphatidylinositol 3-kinase signaling, and the regulation of the MAPK cascade (Figure 13B). Analysis of cellular components indicated that these co-expressed proteins were related to the retromer complex, phosphatidylinositol 3-kinase complex, and membrane protein complexes (Figure 13C). Analysis of molecular functions revealed that the co-expressed proteins were associated with various kinase activities, including phosphatidylinositol-4,5-bisphosphate 3-kinase, 1-phosphatidylinositol-4 phosphate 3-kinase, phosphatidylinositol-3,4-bisphosphate 5-kinase, and insulin receptor binding (Figure 13D). Furthermore, tissue expression analysis indicated a connection between the co-expressed proteins and embryonic cell lines (Figure 13E). The KEGG pathway analysis demonstrated that these proteins were linked to the ErbB signaling pathway and breast cancer (Figure 13F). Additionally, Wiki pathways revealed associations between inflammation, resolvin E1, and resolvin D1 signaling pathways, which promote the resolution of inflammation, as well as the ErbB signaling pathway (Figure 13G). These findings suggest that VPS26A is positioned within a highly interconnected network of signaling and trafficking proteins, many of which have been implicated in cancer progression, inflammation, and cellular metabolism.

## 4. Discussion

Liver hepatocellular carcinoma (LIHC), the most common primary malignancy of the liver, remains a major global health challenge because of its high incidence, aggressive clinical behavior, and poor prognosis. Currently, surgical resection is considered the cornerstone of curative therapy for LIHC and is frequently complemented by chemotherapy, radiotherapy, molecular targeted agents, interventional techniques, and traditional medicine in a multidisciplinary setting [71]. Despite progress in comprehensive treatment techniques, the prognosis of LIHC remains poor with a 5-year survival rate of <20%, which is mainly attributed to the high recurrence rate [72,73]. Therefore, identifying potential biomarkers to improve the prognosis of patients with LIHC is necessary. This poor outcome is primarily because of the high rate of tumor recurrence and intrahepatic metastasis following the initial treatment. These challenges highlight the critical unmet need for improved prognostic tools and novel therapeutic targets that can support early diagnosis, predict disease progression, and inform personalized treatment strategies. Our findings highlight the significance of identifying biomarkers that can facilitate early diagnosis, monitor treatment responses, and predict clinical outcomes in patients with LIHC.

In recent years, the retromer complex has been documented to exhibit a wide array of functions. It is involved in receptor recycling, endosomal tubule dynamics, and the modulation of the actin cytoskeleton [74,75]. This retromer has been implicated in apoptosis [76], mitochondrial membrane dynamics, and Parkinson’s disease [77,78]. Our investigation underscores that VPS26A is a critical component of the retromer complex, which plays a crucial role in endosomal protein sorting and receptor recycling. This positions VPS26A as a potential biomarker for LIHC. Although alterations in other retromer components, such as VPS35 and VPS29, have been associated with cancer and neurodegenerative diseases [79], the role of VPS26A in malignancies, particularly LIHC, has not been thoroughly examined.

In the present study, we evaluated the potential prognostic value of VPS26A by analyzing its association with clinicopathological features and TIICs in LIHC. VPS26A has been identified as a potential prognostic gene; however, its expression and functional roles in LIHC remain unclear. In this study, bioinformatics analyses using various databases demonstrated that both the mRNA and protein expression levels of VPS26A were significantly elevated in LIHC. Furthermore, VPS26A expression was found to correlate with the histological type and tumor stage simplified in LIHC. Prognostic analyses also demonstrated that VPS26A expression had great value for LIHC diagnosis and that VPS26A upregulation was associated with poor prognosis in LIHC. These findings suggest that VPS26A may serve as a valuable biomarker for LIHC diagnosis and prognosis. Notably, combining VPS26A with conventional biomarkers such as alpha-fetoprotein (AFP) could improve diagnostic sensitivity and specificity, particularly in early-stage LIHC or in AFP-negative patients. This combinatorial approach may enhance the clinical applicability of VPS26A and contribute to the development of multi-marker-based precision diagnostic strategies.

The immune system plays a crucial role in modulating cancer progression [18]. TIICs have emerged as a central focus in cancer research [20]. Several studies have highlighted the characteristics of the immune response and its correlation with prognosis [22]. The prognostic relevance of TIICs in LIHC has been notably emphasized. TIICs have been shown to correlate with disease outcomes, leading to the growing recognition of their potential as prognostic markers [80]. Our findings regarding TIICs in LIHC indicated that the expression of VPS26A correlated with various TIICs. These findings suggest that VPS26A may help establish an immune-tolerant TME in advanced LIHC, contributing to poor prognosis and immunotherapy resistance. Its strong link with immunosuppressive TIICs highlights its potential as a biomarker for stratifying patients for immunotherapy.

CNVs are recognized as a defining characteristic of cancer. CNVs have been found to correlate with LIHC stage and prognosis [81,82,83] and are associated with infections caused by hepatitis B or C viruses [84]. Pan-cancer investigations have identified both broad and focal CNV burdens as genomic attributes that can influence tumor immune infiltration and exclusion across various cancer types. Nonetheless, the interpretation of CNVs in LIHC within a diagnostic context remains problematic, primarily owing to the absence of a consensus regarding the relationship between CNVs and clinicopathological characteristics. CNVs and DNA methylation of VPS26A were found to be associated with variations in TIICs.

DNA methylation plays a pivotal role in cancer and several studies have outlined the use of methylated DNA loci as cancer detection markers, focusing mainly on gene promoter markers [85,86]. Some epigenetic markers hold significant promise for the early detection of cancers because they play a crucial role in the initiation of carcinogenic pathways [87,88]. Consequently, epigenetic biomarkers show considerable potential for broad application as early diagnostic indicators. Our investigation revealed that the promoter hypomethylation of VPS26A was prevalent in LIHC and was significantly correlated with elevated gene expression. Importantly, this hypomethylation pattern appeared to be influenced by various clinicopathological parameters, including race, age, tumor stage, and tumor grade. These results reinforce the hypothesis that the epigenetic deregulation of VPS26A represents an early and progressive event in LIHC pathogenesis. Site-specific methylation analysis of VPS26A revealed its prognostic significance. Both hypermethylated and hypomethylated probes located within the promoter and exon regions of VPS26A were associated with poor survival. Hypermethylated probes such as cg14870128, cg21830413, and cg23345864 were significantly correlated with an elevated risk of mortality. Similarly, hypomethylated probes such as cg04149295 and cg07815385 were associated with poor prognosis.

Recent advancements in our understanding of the molecular mechanisms underlying hepatocarcinogenesis coupled with rapid progress in diagnostic methodologies have led to the approval of several therapeutic agents for advanced LIHC. Drug sensitivity assays have been effectively employed in assay-guided chemotherapy for specific malignancies. However, the development of resistance to antineoplastic therapies driven by genetic mutations and various nongenetic factors presents a significant challenge that limits treatment efficacy. This study examined the association between VPS26A expression and drug response in LIHC and found a positive correlation between VPS26A expression and sensitivity to various anticancer agents. Specifically, compounds such as AR-42, belinostat, CAY10603, CUDC-101, FK866, I-BET-762, methotrexate, navitoclax, OSI-027, PHA-793887, OU-103, QL-X-138, tubastatin, UNC0638, vorinostat, and XMD13-2 showed significant positive correlations with VPS26A expression. Conversely, a negative correlation was identified between VPS26A expression and sensitivity to drugs like17-AAG, afatinib, bleomycin, bosutinib, dasatinib, docetaxel, epothilone B, FTI-277, GSK1904529A, JNK inhibitor VIII, PD-0325901, RO-3306, trametinib, and XAV939. These findings suggest that VPS26A plays a crucial role in modulating cellular responses to a diverse array of anticancer agents, underscoring its potential as a predictive biomarker of drug sensitivity in LIHC.

Furthermore, we explored the influence of chemical compounds on the expression of VPS26A. The investigation identified 82 chemicals associated with VPS26A, of which 59 were predicted to enhance its expression, and 23 were linked to its downregulation. Furthermore, the top 20 gene–chemical interactions involving VPS26A were identified, revealing significant correlations with several genes. Notably, VPS26A was strongly associated with genes such as VPS35, GCN1, VTI1B, UFM1, and VPS29. These findings suggest that VPS26A is part of a chemically responsive network that includes key components involved in vesicular trafficking, stress response, and protein sorting. Furthermore, we conducted a systematic examination of the chemical interaction landscape of VPS26A in LIHC, leveraging data from the CTD to identify agents that may affect VPS26A expression. The findings indicated the presence of 82 chemicals with predicted regulatory effects on VPS26A, comprising 59 compounds that upregulate and 23 agents that downregulate VPS26A expression. These interactions provide novel insights into the chemically modifiable characteristics of VPS26A, suggesting its potential involvement in broader regulatory networks that may influence tumor progression and treatment responses in LIHC.

Genes exhibiting strong positive correlations with VPS26A, including DDX50, AP3M1, SMNDC1, RUFY2, and PPP3BX, have been recognized for their roles in RNA metabolism, vesicle transport, and cell division. In contrast, the genes with negative correlations, such as ECHDC2, DHRS3, and AKR7L, are primarily associated with metabolic detoxification and redox regulation. The genes co-expressed with VPS26A are significantly implicated in G2/M phase transition, RNA localization, chromosomal segregation, and spindle organization, all of which are critical for cell proliferation and genomic stability. Furthermore, enrichment analyses of KEGG pathways, including spliceosomes, RNA transport, Wnt signaling, and Hedgehog signaling, suggest that VPS26A may influence oncogenesis by modulating the transcriptional machinery and developmental signaling pathways. Findings from the co-expression and enrichment analyses indicate that VPS26A occupies a central position within the transcriptional network that regulates critical oncogenic processes in LIHC.

We investigated the prognostic significance of genes co-expressed with VPS26A in patients with LIHC. Specifically, among the genes that positively correlated with VPS26A, 31 were associated with a high HR for OS, whereas 13 showed a high HR for DFS. In contrast, 10 of these genes were associated with a low HR for OS and 13 genes showed a low HR for DFS. These findings suggest that VPS26A and its positively correlated gene set are associated with poor prognosis in patients with LIHC.

A gene–gene interaction network was constructed to investigate the functional associations of VPS35, VPS29, VPS26B, and SNX3 with VPS26A. These genes were associated with key cellular processes, including the regulation of autophagy, macroautophagy, endosomal transport, cytosolic transport, and vesicle-mediated transport to the plasma membrane. Their functional convergence highlights their central roles in maintaining intracellular trafficking and homeostasis. These findings provide valuable insights into the molecular framework governing vesicular trafficking, with potential implications for understanding the disorders associated with defects in endosomal transport and autophagy.

MicroRNAs (miRNAs) have been implicated in both the promotion of tumorigenesis and the inhibition of tumor progression, as evidenced in numerous studies. Additionally, the role of miRNAs in tumor prognosis has been well-documented [89]. Research has demonstrated that miRNAs exhibit aberrant expression patterns in LIHC and contribute to the growth, development, and metastasis of this malignancy by functioning as either oncogenes or tumor suppressors. Despite these findings, there remains a lack of validated prognostic models for predicting patient outcomes and guiding treatment strategies for LIHC. Consequently, there is an urgent need to develop an miRNA-based prognostic model for patients with LIHC, which could facilitate accurate prognostication and enable targeted therapies aimed at enhancing overall survival. To investigate the regulatory roles and prognostic significance of miRNAs that target genes associated with VPS26A in LIHC, 61 miRNAs that simultaneously target multiple VPS26A-associated genes were identified, indicating a high level of shared regulatory potential. Among the 61 miRNAs, 15 were significantly correlated with survival outcomes in patients with LIHC. Notably, hsa-miR-302b-3p, hsa-miR-302c-3p, hsa-miR-940, hsa-miR-132-3p, hsa-miR-1262, hsa-miR-197-3p, and hsa-miR-24-3p were associated with a poor prognosis when highly expressed in LIHC. In contrast, low expression levels of hsa-miR-101-3p and hsa-miR-let-7c-5p were associated with poor survival in LIHC. These findings suggest that miRNAs targeting VPS26A-related gene networks may serve as valuable prognostic biomarkers for LIHC.

The PPI network of VPS26A consisted of 234 edges and 39 nodes. PPI network analysis identified five distinct functional clusters, including ErbB/PI3K-AKT signaling, neurodegenerative processes, oxidative stress, leukodystrophy, and mitochondrial dysfunction. The analysis of biological processes through GO and pathway enrichment analyses, with a focus on proteins associated with PPI, revealed significant enrichment in several biological processes. These processes include the positive regulation of AKT signaling, superoxide metabolism, phosphatidylinositol 3-kinase signaling, and the regulation of the MAPK cascade. Furthermore, the analysis of cellular components underscores the association of co-expressed proteins with the retromer complex, phosphatidylinositol 3-kinase complex, and various membrane protein complexes. These proteins exhibit various kinase activities, such as phosphatidylinositol-4,5-bisphosphate 3-kinase activity and insulin receptor binding. Tissue expression analysis suggests a potential link between these proteins and embryonic cell lines. KEGG pathways revealed associations with the ErbB signaling pathway and breast cancer, while Wiki pathways highlighted connections with inflammation resolution pathways involving resolvins E1 and D1, as well as inflammation-related pathways involving COX2, EGFR, and the ErbB signaling pathway. Collectively, these findings suggest that VPS26A is an integral component of a complex network of signaling and trafficking proteins critical for cancer progression, inflammation, and cellular metabolism.

In summary, our comprehensive investigation identified VPS26A as a potential biomarker with significant diagnostic, prognostic, and therapeutic implications for LIHC. Elevated expression of the VPS26A mRNA and protein in LIHC correlates with adverse clinicopathological characteristics and poor patient outcomes. Its association with immunosuppressive tumor-infiltrating immune cells and promoter hypomethylation suggests that VPS26A may play a role in fostering an immune-tolerant tumor microenvironment. Furthermore, both hypermethylation and hypomethylation patterns of VPS26A are associated with reduced survival rates, highlighting the importance of epigenetic regulation in its oncogenic role. Drug sensitivity analysis revealed that VPS26A expression correlated with diverse responses to various anticancer agents, thereby establishing it as a potential predictive biomarker for treatment stratification. Additionally, data regarding chemical interactions and co-expression networks suggested that VPS26A functions within a broader regulatory system involved in vesicle trafficking, stress response, and oncogenic signaling. Functional enrichment analyses further supported its involvement in critical biological pathways, including cell division, transcriptional regulation, and the PI3K-AKT/ErbB signaling pathway, thereby implicating VPS26A in LIHC pathogenesis at multiple levels. This study also identified a set of miRNAs and protein–protein interactions associated with VPS26A, many of which correlated with poor prognosis, suggesting their potential as additional targets for prognostic evaluation or therapeutic intervention. However, this study is limited to bioinformatics-based analyses, and experimental validation was not performed. Future in vitro and in vivo studies are warranted to verify the functional roles of VPS26A and assess its clinical applicability.

## Figures and Tables

**Table 1:** Clinicopathological characteristics of VPS26A expression using KM in LIHC.

Clinicopathological Characteristics	Overall Survival			Relapse-Free Survival			Progression-Free Survival			Disease-Specific Survival		
	(*n* = 364)			(*n* = 316)			(*n* = 370)			(*n* = 362)		
	*N*	Hazard Ratio	*p*-Value	*N*	Hazard Ratio	*p*-Value	*N*	Hazard Ratio	*p*-Value	*N*	Hazard Ratio	*p*-Value
Sex												
Male	246	1.7 (1.08–2.67)	0.02	210	1.35 (0.91–2.02)	0.13	249	1.39 (0.97–1.99)	0.071	244	1.89 (1.05–3.4)	0.03
Female	118	2.55 (1.39–4.68)	0.0019	106	1.66 (0.92–3.02)	0.09	121	1.52 (0.91–2.54)	0.11	118	2.53 (1.17–5.49)	0.015
Stage												
I	170	2.38 (1.25–4.53)	0.0065	153	1.43 (0.83–2.46)	0.19	171	1.59 (0.97–2.62)	0.066	168	1.99 (0.81–4.87)	0.13
I + II	253	2.34 (1.41–3.89)	0.0007	228	1.71 (1.12–2.61)	0.012	256	1.93 (1.32–2.84)	0.00064	251	2.4 (1.18–4.9)	0.013
II	83	1.72 (0.77–3.85)	0.18	75	1.86 (0.95–3.65)	0.068	85	1.75 (0.96–3.2)	0.064	83	2.48 (0.76–8.07)	0.12
II + III	166	1.51 (0.94–2.43)	0.083	145	1.21 (0.77–1.88)	0.4	170	1.25 (0.84–1.86)	0.27	166	1.53 (0.84–2.78)	0.16
III	83	1.13 (0.63–2.05)	0.68	70	0.89 (0.49–1.62)	0.7	85	0.86 (0.5–1.48)	0.59	83	0.99 (0.48–2.04)	0.99
III + IV	87	1.13 (0.64–2.01)	0.67	70	0.89 (0.49–1.62)	0.7	90	0.89 (0.53–1.51)	0.68	87	1.12 (0.56–2.24)	0.74
IV	4	-	-	0	-	-	5	-	-	3	-	-
Grade												
I	55	1.51 (0.58–3.91)	0.4	45	1.82 (0.67–4.94)	0.23	55	1.64 (0.74–3.61)	0.22	55	1.63 (0.47–5.61)	0.44
II	174	1.59 (0.94–2.69)	0.079	149	1.04 (0.64–1.7)	0.86	177	1.35 (0.87–2.08)	0.18	171	1.32 (0.68–2.58)	0.41
III	118	2.43 (1.29–4.58)	0.0047	107	1.84 (1.06–3.2)	0.028	121	1.68 (1.01–2.79)	0.045	119	3.96 (1.66–9.41)	0.0008
IV	12	-	-	11	-	-	12	-	-	12	-	-
AJCC_T												
I	180	2.4 (1.29–4.45)	0.0042	160	1.46 (0.86–2.48)	0.15	181	1.56 (0.96–2.53)	0.071	178	2.08 (0.91–4.77)	0.076
II	90	1.82 (0.86–3.86)	0.12	80	1.68 (0.89–3.17)	0.11	93	1.47 (0.84–2.56)	0.17	91	2.12 (0.78–5.74)	0.13
III	78	1.19 (0.65–2.18)	0.57	67	0.91 (0.49–1.7)	0.78	80	0.88 (0.5–1.55)	0.67	77	1.23 (0.59–2.56)	0.58
IV	13	-	-	6	-	-	13	-	-	13	-	-
Vascular invasion												
None	203	2.7 (1.54–4.73)	0.0003	175	1.61 (0.99–2.63)	0.052	205	1.8 (1.14–2.83)	0.0099	201	3.1 (1.41–6.8)	0.0031
Micro	90	1.35 (0.62–2.94)	0.45	82	0.91 (0.48–1.71)	0.77	92	0.97 (0.55–1.71)	0.92	90	2.19 (0.68–7.13)	0.18
Macro	16	-	-	14	-	-	16	-	-	14	-	-
Race												
White	181	2.23 (1.38–3.6)	0.00075	147	1.51 (0.95− 2.37)	0.077	184	1.58 (1.07–2.35)	0.022	179	2.58 (1.43–4.66)	0.0012
Asian	155	2 (1.08–3.7)	0.025	145	1.27 (0.76–2.1)	0.36	157	1.17 (0.73–1.88)	0.51	154	1.85 (0.83–4.12)	0.13
Alcohol consumption												
Yes	115	1.49 (0.79–2.82)	0.21	99	1.29 (0.72–2.32)	0.38	117	1.33 (0.79–2.22)	0.28	117	1.73 (0.84–3.6)	0.13
None	202	1.96 (1.21–3.18)	0.0051	183	1.49 (0.95–2.32)	0.079	205	1.39 (0.93–2.08)	0.11	199	1.92 (1.01–3.64)	0.042
Hepatitis virus												
Yes	150	2.04 (1.02–4.08)	0.038	139	1.69 (1.02–2.79)	0.04	153	1.87 (1.16–3)	0.009	151	2.8 (1.1–7.12)	0.024
None	167	2.09 (1.29–3.39)	0.0022	143	1.07 (0.65–1.76)	0.8	169	1.27 (0.82–1.97)	0.28	165	1.96 (1.08–3.57)	0.025

**Table 2:** Prognostic significance of VPS26A expression in various cancer types determined using Prognoscan.

Dataset	Cancer Type	Endpoint	*N*	In(HR_high_/HR_low_)	*p*-Value	HR [95% Cl^low^–Cl^upp^]
GSE4271-GPL96	Brain cancer	Overall Survival	77	−1.26	0.0004	0.25 [0.11–0.53]
GSE31210	Lung cancer	Relapse-Free Survival	204	−0.79	0.0025	0.39 [0.21–0.72]
GSE30929	Soft tissue cancer	Distant-Recurrence-Free Survival	140	−0.86	0.0112	0.35 [0.16–0.79]
jacob-00182-MSK	Lung cancer	Overall Survival	104	0.96	0.0247	3.53 [1.17–10.61]
GSE7390	Breast cancer	Relapse-Free Survival	198	−0.5	0.0258	0.57 [0.35–0.93]
GSE9195	Breast cancer	Relapse-Free Survival	77	15.84	0.0325	4.09 [1.12–14.87]
GSE4412-GPL96	Brain cancer	Overall Survival	74	−1.03	0.035	0.33 [0.12–0.92]
GSE9195	Breast cancer	Distant-Metastasis-Free Survival	77	1.48	0.0408	4.56 [1.07–19.50]

**Table 3:** Correlation between VPS26A expression and TIICs using GSCA in LIHC.

Cancer	Gene	Analysis Type	Cell Type	R	*p*-Value
LIHC	VPS26A	Expression	nTreg	0.23	<0.001
			NKT	0.13	0.008
			iTreg	0.17	0.001
			CD8_naive	0.11	0.018
			MAIT	−0.26	<0.001
			NK	−0.21	<0.001
			Cytotoxic	−0.19	<0.001
			Macrophage	−0.14	0.004
			Gamma_delta	−0.14	0.005
			Tfh	−0.13	0.008
			Monocyte	−0.12	0.02
		CNV	CD4_T	0.15	0.004
			NKT	0.12	0.022
			Monocyte	0.11	0.043
			DC	−0.18	0.001
			Macrophage	−0.16	0.003
		Methylation	Bcell	0.11	0.029
			CD4_T	−0.18	0.001
			iTreg	−0.15	0.003
			CD4_naive	−0.14	0.008

**Table 4:** Correlation between VPS26A expression and promoter methylation using OncoDB in LIHC.

Gene	Probe	Chr	Position	Average of Cancer Sample	Average of Normal Sample	*p*-Value
VPS26A	cg07815385	chr10	69123505	0.07	0.04	<0.001
	cg04362027	chr10	69123545	0.06	0.04	0
	cg04615147	chr10	69123581	0.09	0.06	0
	cg11420031	chr10	69123745	0.03	0.02	<0.001
	cg23345864	chr10	69123774	0.03	0.04	<0.001
	cg02393810	chr10	69123802	0.03	0.03	0.9
	cg06726474	chr10	69123970	0.03	0.03	0.8
	cg21830413	chr10	69124495	0.02	0.02	0.07
	cg19111245	chr10	69124664	0.08	0.06	0
	cg23656443	chr10	69124886	0.06	0.06	0.3
	cg11621586	chr10	69124914	0.03	0.03	0.02
	cg04149295	chr10	69124960	0.05	0.04	<0.001
	cg14870128	chr10	69171556	0.82	0.89	<0.001

**Table 5:** Interactions between VPS26A expression and chemicals in LIHC using the CTD.

Chemical Name	ID	Interaction Actions
2,2′,4,4′-tetrabromodiphenyl ether	C511295	Increases expression
4-(5-benzo (1,3) dioxol-5-yl-4-pyridin-2-yl-1H-imidazol-2-yl) benzamide	C459179	Increases expression
Air pollutants	D000393	Increases expression
Ammonium 2,3,3,3-tetrafluoro-2-(heptafluoropropoxy)-propanoate	C000611729	Increases expression
Aroclors	D001140	Increases expression
Benzene	D001554	Increases expression
Bisphenol A	C006780	Increases expression
Bisphenol A	C006780	Increases expression
Bisphenol AF	C583074	Increases expression
Bisphenol B	C492482	Increases expression
Bisphenol F	C000611646	Increases expression
Cadmium chloride	D019256	Increases expression
Chlordan	D002706	Increases expression
Chloropicrin	C100187	Increases expression
Cobaltous chloride	C018021	Increases expression
Cupric chloride	C029892	Increases expression
Dactinomycin	D003609	Increases expression
Decamethrin	C017180	Increases expression
Diuron	D004237	Increases expression
Dronabinol	D013759	Increases expression
Ethanol	D000431	Increases expression
Finasteride	D018120	Increases expression
Fish oil	D005395	Increases expression
Flutamide	D005485	Increases expression
FR900359	C000607068	Increases expression
Gentamicin	D005839	Increases expression
Glucose	D005947	Increases expression
Hexachlorocyclohexane	D001556	Increases expression
Hydrocarbons, chlorinated	D006843	Increases expression
Hydrogen peroxide	D006861	Increases expression
Ionomycin	D015759	Increases expression
LDN 193189	C554430	Increases expression
Methylparaben	C015358	Increases expression
Nefazodone	C051752	Increases expression
Niclosamide	D009534	Increases expression
Nimesulide	C012655	Increases expression
Nutlin 3	C482205	Increases expression
Oxybenzone	C005290	Increases expression
Particulate matter	D052638	Increases expression
Pentabromodiphenyl ether	C086401	Increases expression
Perfluorohexanesulfonic acid	C471071	Increases expression
Perfluorooctane sulfonic acid	C076994	Increases expression
Phenobarbital	D010634	Increases expression
Phosphinothricin	C003121	Increases expression
Pirinixic acid	C006253	Increases expression
Plant extract	D010936	Increases expression
Resveratrol	D000077185	Increases expression
Sodium arsenite	C017947	Increases expression
Tetradecanoylphorbol acetate	D013755	Increases expression
Theophylline	D013806	Increases expression
Titanium dioxide	C009495	Increases expression
Tobacco smoke pollution	D014028	Increases expression
Toxaphene	D014112	Increases expression
Triphenyl phosphate	C005445	Increases expression
Triptonide	C084079	Increases expression
Valdecoxib	C406224	Increases expression
Valproic acid	D014635	Increases expression
1,2-dimethylhydrazine	D019813	Decreases expression
3-dinitrobenzene	C017906	Decreases expression
4-hydroxyphenyl 4-isopropoxyphenylsulfone	C000613560	Decreases expression
Aflatoxin M1	D016607	Decreases expression
Bisphenol A	C006780	Decreases expression
Bisphenol A	C006780	Decreases expression
Bisphenol F	C000611646	Decreases expression
Bisphenol S	C543008	Decreases expression
Chlorodiphenyl (54% chlorine)	D020111	Decreases expression
Choline	D002794	Decreases expression
Copper sulfate	D019327	Decreases expression
Dicrotophos	C000944	Decreases expression
Fenthion	D005284	Decreases expression
Folic acid	D005492	Decreases expression
Ivermectin	D007559	Decreases expression
Methionine	D008715	Decreases expression
Mocetinostat	C523184	Decreases expression
Pentabrominated diphenyl ether 100	C517827	Decreases expression
Perfluorooctanoic acid	C023036	Decreases expression
Sodium arsenite	C017947	Decreases expression
Temozolomide	D000077204	Decreases expression
Testosterone	D013739	Decreases expression
Titanium dioxide	C009495	Decreases expression

**Table 6:** Interactions between VPS26A-related genes and chemicals in LIHC using CTD.

Gene	Similarity Index	Common Interaction Chemicals
VPS35	0.36	45
GCN1	0.3186	36
VTI1B	0.3056	33
UFM1	0.3053	40
VPS29	0.3016	38
USP10	0.2979	42
FAM98A	0.2929	41
COPB1	0.2901	38
MARS1	0.2897	42
ARFIP1	0.2868	37
SUPV3L1	0.2857	32
LAMTOR5	0.2832	32
SLC33A1	0.2823	35
ALG5	0.2818	31
KCMF1	0.2813	36
PSMD14	0.281	43
NSFL1C	0.2808	41
VARS1	0.2778	40
EIF3B	0.2774	43
PFDN4	0.2773	33

**Table 7:** miRNAs targeting VPS26A-associated genes in LIHC.

VPS26A		VPS35		VPS29		VPS26B		SNX3	
hsa-let-7a-5p	hsa-miR-216a-5p	hsa-let-7a-5p	hsa-miR-141-3p	hsa-let-7a-5p	hsa-miR-221-3p	hsa-let-7a-5p	hsa-miR-33a-5p	hsa-let-7a-5p	hsa-miR-199a-3p
hsa-let-7b-5p	hsa-miR-221-3p	hsa-let-7b-5p	hsa-miR-145-5p	hsa-let-7b-5p	hsa-miR-1-3p	hsa-let-7b-5p	hsa-miR-96-5p	hsa-let-7b-5p	hsa-miR-34a-5p
hsa-let-7c-5p	hsa-miR-222-3p	hsa-let-7c-5p	hsa-miR-152-3p	hsa-let-7c-5p	hsa-miR-124-3p	hsa-let-7c-5p	hsa-miR-29b-3p	hsa-let-7c-5p	hsa-miR-181a-5p
hsa-miR-15a-5p	hsa-miR-132-3p	hsa-miR-15a-5p	hsa-miR-185-5p	hsa-miR-29a-3p	hsa-miR-130a-3p	hsa-miR-15a-5p	hsa-miR-192-5p	hsa-miR-15a-5p	hsa-miR-1-3p
hsa-miR-21-5p	hsa-miR-126-5p	hsa-miR-21-5p	hsa-miR-193a-3p	hsa-miR-92a-3p	hsa-miR-132-3p	hsa-miR-21-5p	hsa-miR-34a-5p	hsa-miR-21-5p	hsa-miR-122-5p
hsa-miR-24-3p	hsa-miR-193a-3p	hsa-miR-24-3p	hsa-miR-194-5p	hsa-miR-96-5p	hsa-miR-29c-3p	hsa-miR-24-3p	hsa-miR-1-3p	hsa-miR-26a-5p	hsa-miR-194-5p
hsa-miR-26a-5p	hsa-miR-195-5p	hsa-miR-26a-5p	hsa-miR-195-5p	hsa-miR-101-3p	hsa-miR-494-3p	hsa-miR-26a-5p	hsa-miR-124-3p	hsa-miR-26b-5p	hsa-miR-155-5p
hsa-miR-26b-5p	hsa-miR-302b-3p	hsa-miR-26b-5p	hsa-miR-200c-3p	hsa-miR-29b-3p	hsa-miR-503-5p	hsa-miR-26b-5p	hsa-miR-193a-3p	hsa-miR-29a-3p	hsa-miR-520f-3p
hsa-miR-31-5p	hsa-miR-302c-3p	hsa-miR-28-5p	hsa-miR-155-5p	hsa-miR-34a-5p	hsa-miR-1262	hsa-miR-28-5p	hsa-miR-194-5p	hsa-miR-31-5p	hsa-miR-192-3p
hsa-miR-33a-5p	hsa-miR-519d-3p	hsa-miR-29a-3p	hsa-miR-29c-3p			hsa-miR-29a-3p	hsa-miR-29c-3p	hsa-miR-92a-3p	hsa-miR-129-2-3p
hsa-miR-92a-3p	hsa-miR-503-5p	hsa-miR-31-5p	hsa-miR-200a-3p			hsa-miR-31-5p	hsa-miR-34c-5p	hsa-miR-29b-3p	
hsa-miR-96-5p	hsa-miR-769-5p	hsa-miR-33a-5p	hsa-miR-34c-5p						
hsa-miR-101-3p	hsa-miR-766-3p	hsa-miR-92a-3p	hsa-miR-302b-3p						
hsa-miR-199a-3p	hsa-miR-145-3p	hsa-miR-101-3p	hsa-miR-429						
hsa-miR-34a-5p	hsa-miR-33b-3p	hsa-miR-29b-3p	hsa-miR-449a						
hsa-miR-181a-5p		hsa-miR-197-3p	hsa-miR-493-3p						
		hsa-miR-34a-5p	hsa-miR-769-5p						
		hsa-miR-181a-5p	hsa-miR-766-3p						
		hsa-miR-200b-3p	hsa-miR-145-3p						
		hsa-miR-124-3p	hsa-miR-499a-3p						
		hsa-miR-130a-3p	hsa-miR-940						

## Data Availability

All data are available on reasonable request to the corresponding author.
